# miR-129-5p targets Wnt5a to block PKC/ERK/NF-κB and JNK pathways in glioblastoma

**DOI:** 10.1038/s41419-018-0343-1

**Published:** 2018-03-12

**Authors:** Ailiang Zeng, Jianxing Yin, Yan Li, Rui Li, Zheng Wang, Xu Zhou, Xin Jin, Feng Shen, Wei Yan, Yongping You

**Affiliations:** 1 Department of Neurosurgery, The First Affiliated Hospital of Nanjing Medical University, 210029 Nanjing, PR China; 2 Beijing Neurosurgical Institute, Capital Medical University, 100050 Beijing, PR China

## Abstract

Therapeutic application of microRNAs (miRNAs) in Wnt-driven glioma has been valuable; however, their specific roles and mechanisms have not been completely investigated. Real-time quantitative PCR (RT-qPCR) was used to analyse the expression of microRNA-129-5p (miR-129-5p) in human glioma samples. Cell-Counting Kit 8 (CCK-8), flow cytometry, EdU, angiogenesis, Transwell invasion, wound healing, in vitro 3D migration and neurosphere formation assays were employed to assess the role of miR-129-5p in glioblastoma multiforme (GBM) cells. Moreover, we performed the luciferase reporter assay and the RNA-ChIP (chromatin immunoprecipitation) assay to confirm whether Wnt5a was a direct target of miR-129-5p. We also confirmed the correlation between the expression profile of miR-129-5p and Wnt5a in glioma patients from the Chinese Glioma Genome Atlas (CGGA) and investigated the overall survival of GBM patients using two data sets, namely, TCGA and GSE16011, according to their Wnt5a expression status. MiR-129-5p expression levels were downregulated and inversely correlated with Wnt5a expression levels in CGGA glioma patients. Restored expression of miR-129-5p blocked GBM cell proliferation, invasion, migration, angiogenesis, neurosphere formation and resistance to temozolomide. We reported that miR-129-5p directly targeted Wnt5a in glioma. Furthermore, we observed that overexpression of miR-129-5p inhibited the expression of Wnt5a, thus blocking the protein kinase C(PKC)/ERK/NF-κB and JNK pathways. Inhibiting Wnt5a rescued the effects of miR-129-5p loss and increased Wnt5a expression was associated with reduced overall survival of GBM patients. We also demonstrated the inhibitory effect of miR-129-5p on tumour growth in GBM using an in vivo model. The miR-129-5p/Wnt5a-axis-mediated PKC/ERK/NF-κB and JNK pathways have therapeutic potential in GBM treatment.

## Introduction

Gliomas represent the most common primary brain tumours in adults and glioblastoma multiforme (GBM) has been categorised as a WHO grade IV disease^1^. Despite recent advances in its diagnosis and the combination of surgery, chemotherapy and radiation therapy for its treatment, the prognosis for GBM remains poor^1^. Temozolomide (TMZ), a DNA alkylating antineoplastic drug is currently used as first-line therapy. However, a major impediment to effective drug treatment is the development of TMZ resistance.

microRNAs (miRNAs) have been demonstrated to play central roles in the development, progression and recurrence of human cancers. Particularly, miR-129-5p has been demonstrated to be downregulated in multiple types of cancers^2–9^. Recently, miR-129-5p was observed to be downregulated in GBM tissues compared to that in adjacent non-tumourous tissues^10^. Furthermore, the role of miR-129-5p in regulating proliferation and invasion of GBMs was demonstrated in U87 cell lines, in vitro^10^. However, there is a need to characterise the landscape of miR-129-5p expression in all malignant gliomas. Explicit molecular mechanisms of miR-129-5p in GBM need to be fully explored in multiple GBM cell lineages, including primary GBM cells and GBM stem cells (GSCs).

The non-canonical Wnt molecule, Wnt5a, is a representative ligand of non-canonical Wnt signalling^11,12^. The planar cell polarity (PCP) and Wnt/Ca^2+^ pathways are the best characterised among the Wnt5a downstream pathways and are involved in cell physiology and development of various cancers^12,13^. The PCP pathway modulates cell polarity and morphogenetic movements through the activation of c-Jun N-terminal protein kinase (JNK)^12–14^. The Wnt/Ca^2+^ pathway modulates cell adhesion and motility through the activation of phospholipase C, PKC and calmodulin-dependent protein Kinase II^12–14^. Wnt5a expression is higher in glioma than that in normal brain and is correlated with WHO histological grade progression^11,14–17^. The role of Wnt5a in promoting proliferation and migration of cells has been demonstrated in gliomas, in vitro^11,12,16–18^. Recently, it was also demonstrated that Wnt5a functions as a master regulator of the proliferative and invasive capacity of GSCs in intracranial xenograft models^11,19,20^. However, the upstream modulators of Wnt5a and networks of downstream signalling that bestow the malignant phenotype on GBM cells remain undetermined.

Here, we determine whether miR-129-5p directly represses Wnt5a expression, which inactivates non-canonical Wnt signalling and leads to the subsequent inhibition of GBM cell proliferation, angiogenesis, epithelial/mesenchymal transition (EMT), invasion, migration, neurosphere formation, chemoresistance and in vivo tumour growth. In an exploration of the mechanism of miR-125-5p action, we demonstrate that dysregulation of miR-129-5p/Wnt5a signalling activates the PKC/ERK/NF-κB and JNK pathways, leading to a more malignant phenotype and resistance to TMZ.

## Results

### miR-129-5p expression is downregulated in glioma

**Fig. 1:**
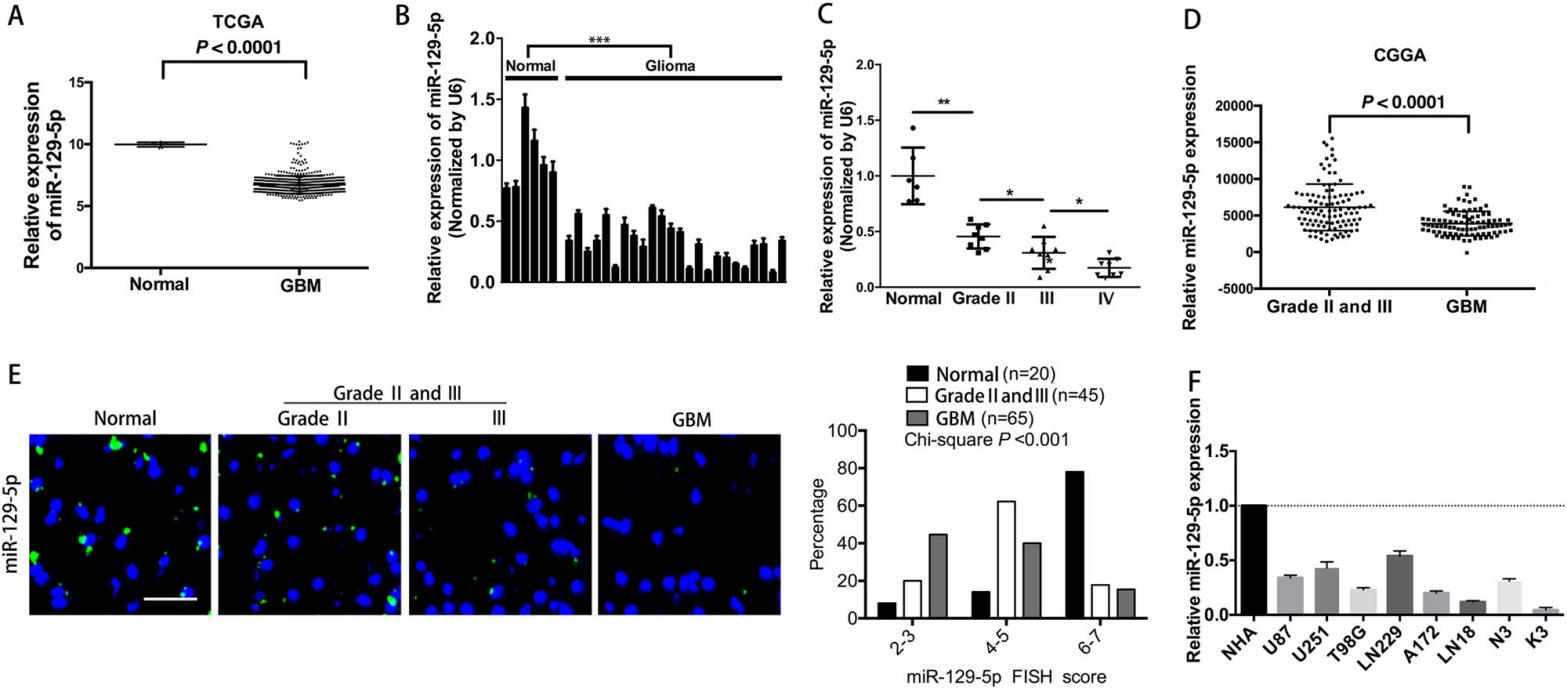
miR-129-5p is downregulated in glioma patient specimens. **a** Relative expression of miR-129-5p was determined in the TCGA data set (normal brain tissues vs. GBM tissues); *P* < 0.0001. **b** Relative miR-129-5p expression was analysed in 6 normal brain tissues and 24 glioma tissues (8 grade II, 8 grade III and 8 grade IV) (six replicates per sample, three independent experiments per sample). **c** Twenty-four glioma tissues were grouped into 3 groups: 8 WHO grade II, 8 grade III and 8 GBMs (2 Classical, 2 mesenchymal, 1 neural and 3 proneural GBMs). Relative expression of miR-129-5p in normal brain tissues and 3 different grades of glioma samples was determined. *P* < 0.0001. **d** Expression levels of miR-129-5p in the Chinese Glioma Genome Atlas (CGGA) cohort of human malignant glioma patients (GBM vs. Grades II and III). **e** Representative images of FISH analysis of miR-129-5p expression in normal brain and WHO grade II, grade III and grade IV glioma (GBM) tissues. Scale bar, 100 μm. Histograms of miR-129-5p FISH scores in normal brain (*n* = 20), grade II and III (*n* = 45) and GBM specimens (*n* = 65) (miR-129-5p loss was defined by a FISH score of 2–3) (Chi-square test, *P* < 0.001) (three replicates per sample, three independent experiments per sample). **f** Expression of miR-129-5p was analysed in NHAs and eight GBM cells (six replicates per group, three independent experiments per group). Data are expressed as the mean ± s.e.m

We first analysed miR-129-5p levels in data from 491 GBM patients using the TCGA database. miR-129-5p expression of GBMs was significantly downregulated compared to that of matched normal brain tissues (Fig. 1a). We also quantified the expression of miR-129-5p in 24 glioma samples, divided into three groups with different grades and 6 normal brain tissue samples (Fig. 1b). miR-129-5p levels were downregulated in these 3 glioma groups compared to those in the normal brain group, and the grade IV gliomas (GBMs) harboured the lowest expression of miR-129-5p (Fig. 1c). miR-129-5p levels were significantly decreased in GBM compared to those in Grade II and III tissues in the CGGA data set (Fig. 1d). Moreover, fluorescence in situ hybridisation (FISH) analysis confirmed a higher rate of miR-129-5p loss in GBM tissues (45%) than that in grade II and III tissues (20%) and normal brain tissues (8%) (Fig. 1e). GBM cells expressed lower miR-129-5p levels compared to normal human astrocytes (NHAs) (Fig. 1f). Our results indicated that miR-129-5p expression is downregulated in glioma.

### Wnt5a is a direct target of miR-129-5p

**Fig. 2:**
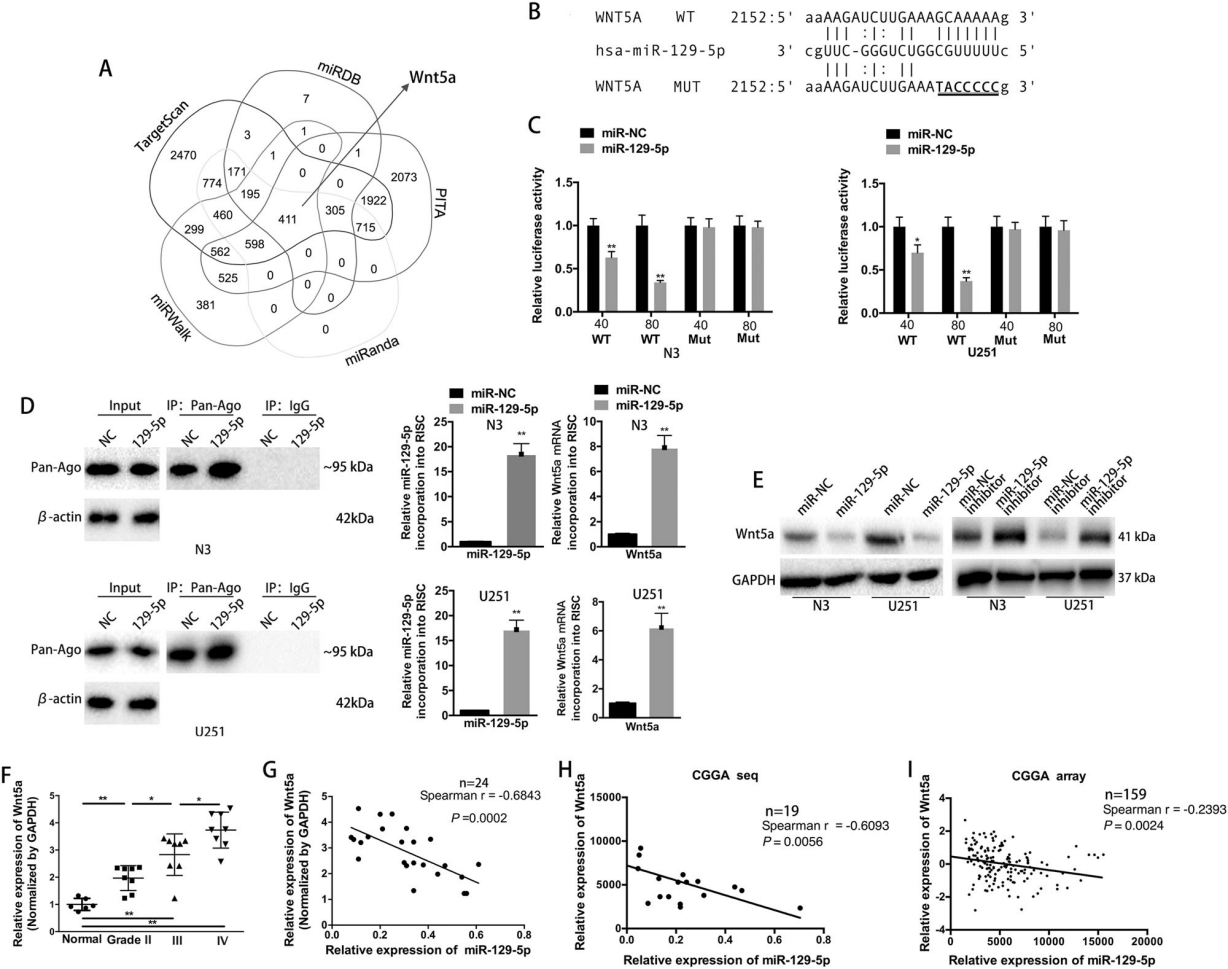
miR-129-5p directly targets Wnt5a in GBM cells. **a** Venn diagram displaying miR-129-5p computationally predicted to target Wnt5a by five different prediction algorithms: TargetScan, miRDB, PITA, miRanda and miRWalk. **b** Predicted miR-129-5p target sequence in Wnt5a-3′ UTRs. Target sequences of Wnt5a-3′ UTRs were mutated. **c** Luciferase assay of cells transfected with Wnt5a-3′ UTR-WT or Wnt5a-3′ UTR-MUT reporter together with 40 nM or 80 nM miR-129-5p or miR-NC mimic (six replicates per group, three independent experiments per group). **d** Immunoprecipitation (left) of the Ago2/RISC (RNA-induced silencing complex) using the Pan-Ago2 antibody in N3 or U251 cells overexpressing miR-NC or miR-129-5p. IgG was used as a negative control and β-actin was used as an internal control. RT-qPCR analysis (middle) of miR-151a incorporated into RISC in N3 or U251 cells overexpressing miR-129-5p compared to the levels in the control. U6 RNA was used as an internal control. RT-qPCR (right) of Wnt5a incorporated into RISC in N3 or U251 cells overexpressing miR-129-5p. GAPDH RNA was used as an internal control (three replicates per group, three independent experiments per group). **e** Western blot analysis indicated that Wnt5a expression levels were decreased in cells with miR-129-5p mimic overexpression, but increased in cells treated with a miR-129-5p inhibitor. GAPDH was used as the loading control (three replicates per group, three independent experiments per group). **f** Wnt5a expression levels in 6 normal brain tissues and 24 glioma specimens (8 glioma tissues in each group: WHO grade II, grade III and grade IV) were examined by RT-qPCR (three replicates per group, three independent experiments per group). **g** Spearman correlation analysis was employed to confirm the correlations between the Wnt5a and miR-129-5p expression levels in 24 human glioma specimens. Spearman *r* = −0.6843, *P* = 0.0002. **h** and **i** Spearman correlation test of miR-129-5p expression with Wnt5a expression, as indicated by the CGGA data sets; Spearman *r* = −0.6093, *P* = 0.0056 and Spearman *r* = −0.2393, *P* = 0.0024, respectively. Data are expressed as the mean ± s.e.m

To identify the mechanism of miR-129-5p in glioma, we applied bioinformatic algorithms to predict potential target genes. As predicted by TargetScan, miRDB, PITA, miRanda and miRWalk, there was complementarity between miR-129-5p and Wnt5a-3′ UTR (Fig. 2a). A previous study also demonstrated that miR-129-5p binds to the 3′ UTR of Wnt5a in human vascular smooth muscle cells^21^. However, miRNAs may suppress their targets in a context-dependent manner^22^. Luciferase expression assays were performed to functionally verify whether miR-129-5p directly targets Wnt5a in GBM cells (Fig. 2b). Wild-type miR-129-5p binding sites of the Wnt5a-3′ UTR led to decreased luciferase activity relative to mutated binding sites in N3 and U251 cells co-transfected with a miR-129-5p mimic (Fig. 2c). RNA-ChIP analysis was also employed to selectively detect Wnt5a mRNA abundance in the Ago2/RNA-induced silencing complex (RISC) complex after miR-129-5p overexpression, confirmed by RT-qPCR (Supplementary Fig. 1A and Fig. 2d, left and middle). Enrichment in the levels of Wnt5a that was incorporated into RISC was observed in miR-129-5p-overexpressing cells (Fig. 2d, right). Cells transiently overexpressing the miR-129-5p mimic showed reduced the expression of Wnt5a, while silencing miR-129-5p (by miR-129-5p inhibitor) upregulated the expression of Wnt5a (Fig. 2e). The expression of Wnt5a mRNA was significantly higher in 24 gliomas than that in 6 normal brain tissues (Fig. 2f). As shown in Fig. 2g, the expression of Wnt5a and miR-129-5p in 24 glioma specimens were observed to be inversely correlated. The Spearman test of correlation of miR-129-5p expression with Wnt5a transcript expression from both CGGA mRNA sequences and array data sets also revealed a statistically significant inverse correlation between miR-129-5p and Wnt5a expression levels (Fig. 2h, i). GBM cell lines also expressed higher levels of Wnt5a compared to NHAs (Supplementary Fig. 1B). All data indicated that Wnt5a is a direct target of miR-129-5p.

### miR-129-5p overexpression decreases cell proliferation and angiogenesis of GBMs by targeting Wnt5a

**Fig. 3:**
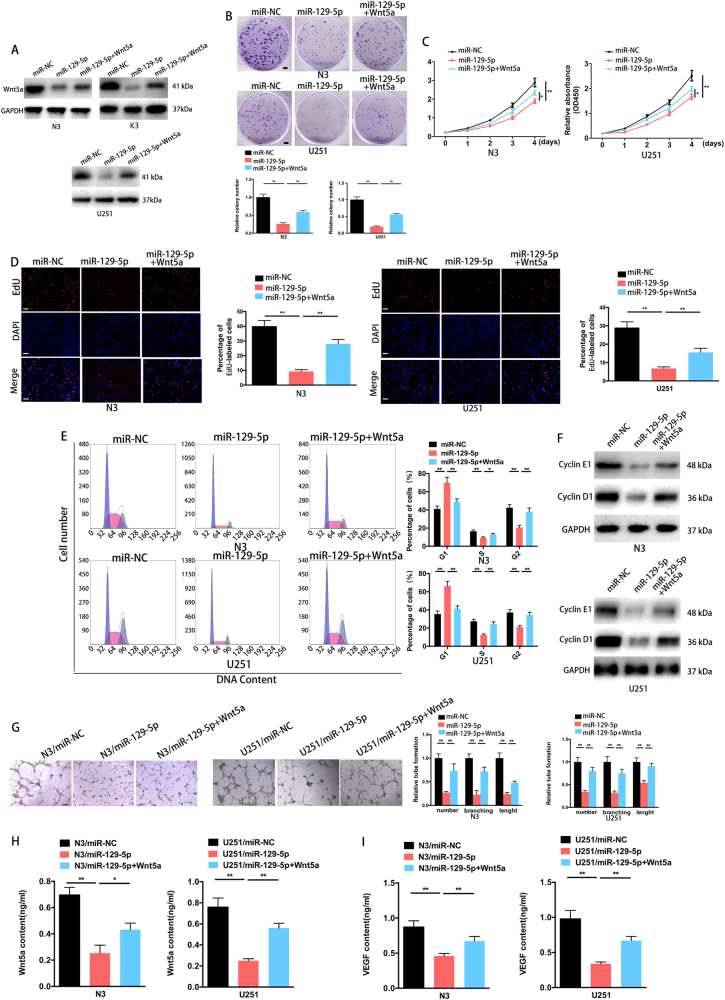
miR-129-5p overexpression inhibits cell proliferation and angiogenesis in GBM by targeting Wnt5a . **a** N3/miR-129-5p, K3/miR-129-5p and U251/miR-129-5p cells were transfected with the Wnt5a-plasmid vector, followed by western blot analysis of Wnt5a transcripts (three replicates per group, three independent experiments per group). GAPDH served as the loading control. **b** Colony formation ability of the miR-NC- or miR-129-5p-transfected N3 or U251 cells without transfection or transfected with the pcDNA3.1-Wnt5a plasmid (Wnt5a) (six replicates per group, three independent experiments per group). Scale bar, 2.5 mm. **c** Overexpression of miR-129-5p arrested cell proliferation; however, this was rescued upon coexpression of exogenous Wnt5a in N3 and U251 cells (six replicates per group, three independent experiments per group). **d** EdU analysis of miR-NC, miR-129-5p or miR-129-5p plus Wnt5a-transfected N3 and U251 cells (six replicates per group, three independent experiments per group). Scale bar, 100 μm. **e** Cell-cycle assay of N3 and U251 GBM cells 3 days after transfection with miR-NC, miR-129-5p or miR-129-5p plus Wnt5a (six replicates per group, three independent experiments per group). **f** Western blot analysis indicated the regulation of the cell-cycle-regulatory proteins cyclin E1 and cyclin D1 in miR-NC, miR-129-5p or miR-129-5p mimic plus Wnt5a-transfected N3 or U251 cells. GAPDH was used as the loading control (three replicates per group, three independent experiments per group). **g** Representative images and quantification of HBMVECs cultured on Matrigel-coated 96-well plates using conditioned medium from the indicated cells (six replicates per group, three independent experiments per group). Scale bar, 200 μm. Data are expressed as the mean ± s.e.m. **h**–**i** Quantitative determination of Wnt5a and VEGF levels in supernatants from N3 or U251 cells with indicated treatment by ELISA. (six replicates per group, three independent experiments per group). Data are expressed as the mean ± s.e.m

To study the influence of miR-129-5p on cellular proliferation, we transfected the primary GBM N3 cell and U251 cell with miR-NC (N3/miR-NC and U251/miR-NC) or a miR-129-5p mimic (N3/miR-129-5p and U251/miR-129-5p). As expected, the colony formation and CCK-8 assays demonstrated that miR-129-5p upregulation significantly inhibited colony formation (Fig. 3b) and the proliferation rate of N3 and U251 cells (Fig. 3c). We then tested the effect of Wnt5a upregulation by using a Wnt5a plasmid (not including the 3′ UTR)-based strategy in N3/miR-129-5p and U251/miR-129-5p cells. Wnt5a protein levels were increased in Wnt5a-co-transfected N3/miR-129-5p and U251/miR-129-5p cells (N3/miR-129-5p + Wnt5a and U251/miR-129-5p + Wnt5a) over N3/miR-129-5p and U251/miR-129-5p cells (Fig. 3a). Enforced Wnt5a expression abolished the inhibited colony formation and proliferation caused by miR-129-5p overexpression (Fig. 3b, c). Moreover, EdU assays demonstrated that miR-129-5p-overexpressing N3 and U251 cells exhibited a significant decrease in the number of EdU-positive cells compared to those in the control cells (Fig. 3d). Flow cytometry demonstrated that miR-129-5p overexpression induced cell-cycle arrest and elevated the percentage of cells in the G1 phase at 72 h post-transfection while decreasing the percentage of cells in the S phase in both N3 and U251 cells (Fig. 3e). Consistent with previous studies reporting that cyclin D1 and cyclin E1 accelerated the G1/S phase transition in glioma cells^23^, cyclin E1 and cyclin D1 expression levels were decreased following enforced miR-129-5p expression (Fig. 3f). Human brain microvessel endothelial cells (HBMVECs) cultured in conditioned medium obtained from miR-129-5p-overexpressing GBM cells displayed fewer vessels and branches and a shorter tube length (Fig. 3g). Quantitative determination by ELISA in miR-129-5p-overexpressing GBM cell supernatants demonstrated impaired Wnt5a and VEGF secretion compared to that in control cells (Fig. 3h, i). Enforced Wnt5a expression in miR-129-5p-overexpressing cells abolished the effect of the inhibited proliferation, G1/S phase transition and angiogenesis functions of miR-129-5p (Fig. 3d–i). Thus, miR-129-5p overexpression dampens GBM cell proliferation and angiogenesis by targeting Wnt5a.

### miR-129-5p suppresses invasion and migration in human GBM cells by targeting Wnt5a

**Fig. 4:**
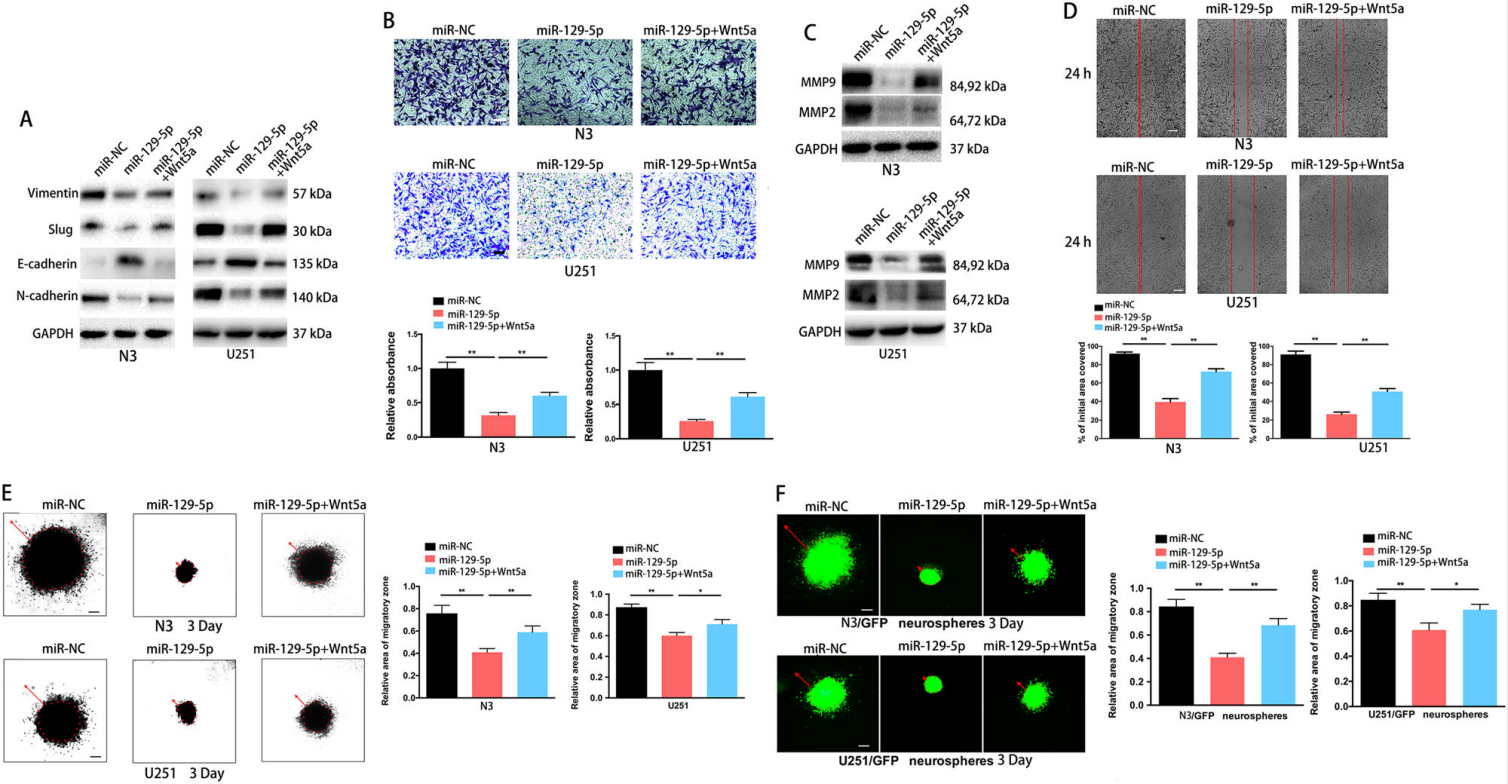
miR-129-5p overexpression blocks invasion and migration of GBM cells by targeting Wnt5a . **a** EMT-associated proteins in miR-NC, miR-129-5p or miR-129-5p mimic plus Wnt5a-transfected N3 or U251 cells were determined by western blotting. GAPDH was used as the loading control (three replicates per group, three independent experiments per group). **b** Effect of miR-129-5p overexpression on miR-NC, miR-129-5p or miR-129-5p plus Wnt5a-transfected GBM cell invasion was examined by a Matrigel invasion assay (six replicates per group, three independent experiments per group). Scale bar, 100 μm. **c** Protein levels of MMP9 and MMP2 in miR-NC, miR-129-5p or miR-129-5p mimic plus Wnt5a-transfected N3 or U251 cells (three replicates per group, three independent experiments per group). GAPDH was used as the loading control. **d** Migration of GBM cells was monitored by a wound-healing assay. Representative images of GBM cells transfected with either miR-NC or miR-129-5p (six replicates per group, three independent experiments per group). miR-129-5p-mediated GBM migration was rescued by forced coexpression of Wnt5a. Scale bar, 200 μm. **e** Results of GBM cell migration were validated by a 3D spheroid migration assays (six replicates per group, three independent experiments per group). Scale bar, 200 μm. **f** Migration of N3/GFP neurospheres and U251/GFP neurospheres was monitored by 3D spheroid migration assays. Representative images and quantification of spheroid migration of neurospheres enriched from miR-NC, miR-129-5p or miR-129-5p plus Wnt5a-transfected GBM cells (six replicates per group, three independent experiments per group). Scale bar, 100 μm. Data are expressed as the mean ± s.e.m

Next, we investigated the effect of miR-129-5p overexpression on EMT in GBM cells. Expression levels of the mesenchymal markers vimentin, slug and N-cadherin were downregulated, while those of E-cadherin were upregulated in miR-129-5p-overexpressing N3 or U251 cells (Fig. 4a). Transwell assays demonstrated that restoration of miR-129-5p dramatically inhibited the normally strong invasive capacity of N3 and U251 cells (Fig. 4b). To investigate the mechanism whereby miR-129-5p inhibited GBM cell invasion, we determined the expression of the invasion-associated molecules, matrix-metalloproteinase (MMP) 9 and MMP2. MMP9 and MMP2 protein levels were markedly decreased upon miR-129-5p overexpression (Fig. 4c). Enforced expression of Wnt5a restored the EMT change and invasion that was inhibited by miR-129-5p (Fig. 4a–c). Simultaneously, wound-healing assays demonstrated that miR-129-5p-overexpressing cells had decreased migration compared to that of cells transfected with miR-NC (Fig. 4d). Meanwhile, miR-129-5p overexpression inhibited cell infiltration in a 3D collagen matrix^24,25^ and miR-129-5p-overexpressing cells demonstrated a less invasive morphology compared to that of the control cells (Fig. 4e). GFP-expressing N3 and U251 neurospheres (N3/GFP and U251/GFP neurospheres) seeded in a 3D collagen matrix (Fig. 4f) showed similar patterns as those observed in the invasion assays. Enforced expression of Wnt5a reversed miR-129-5p-mediated suppression of cell migration (Fig. 4e, f). Thus, overexpression of miR-129-5p suppresses GBM cell invasion and migration by targeting Wnt5a.

### Overexpression of miR-129-5p inhibits neurosphere formation and confers chemosensitivity to TMZ by targeting Wnt5a

**Fig. 5:**
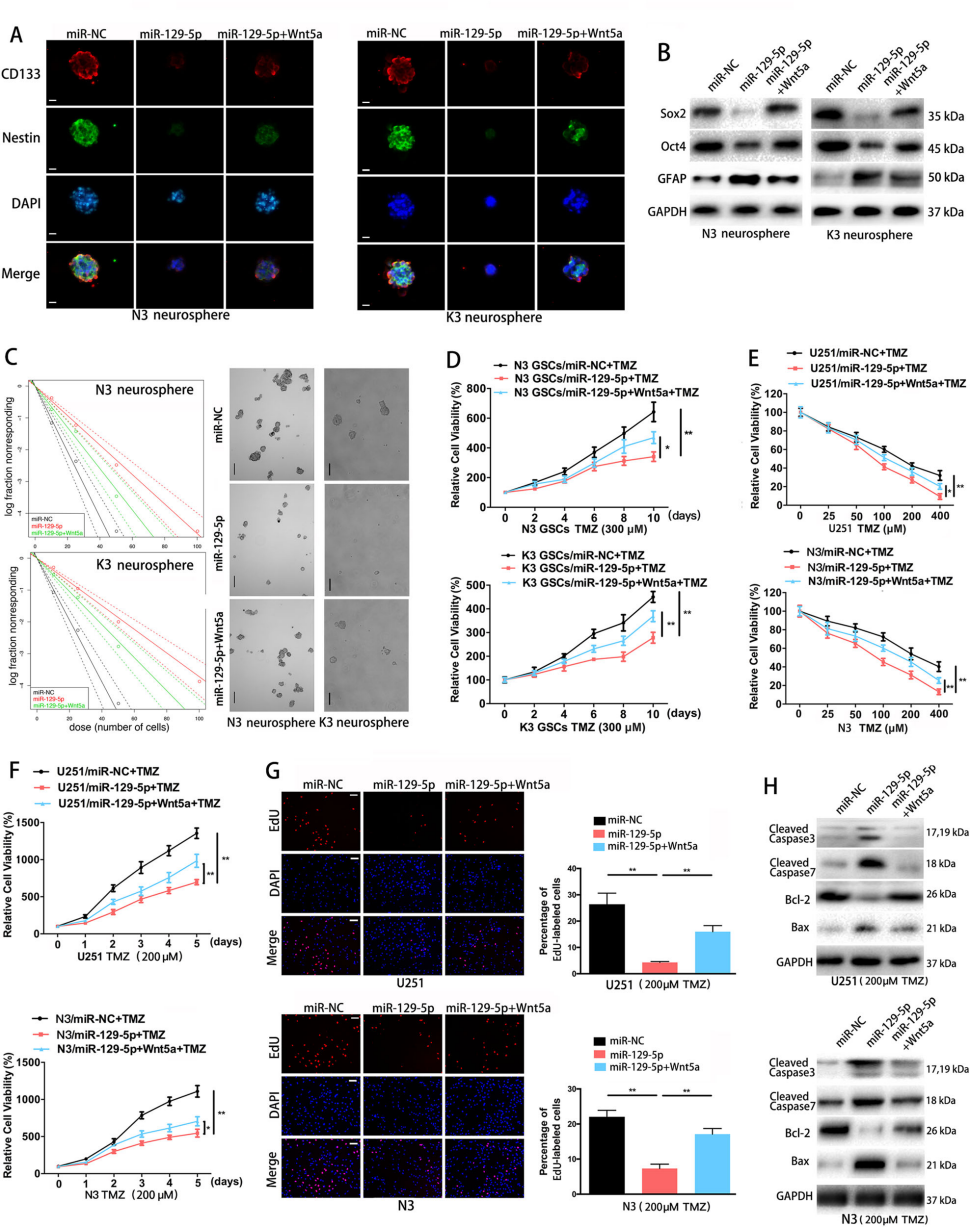
Overexpression of miR-129-5p in GBM cells inhibits neurosphere formation and confers chemosensitivity to TMZ by targeting Wnt5a. **a** Representative immunofluorescence staining images of CD133 (red) and Nestin (green) in stem-like N3 and K3 neurospheres. Scale bar, 20 μm. **b**Western blot analysis of Sox2, Oct4 and GFAP in N3 and K3 neurospheres transfected with miR-NC, miR-129-5p or miR-129-5p plus Wnt5a (three replicates per group, three independent experiments per group). GAPDH was used as a loading control. **c** Limiting dilution assay (LDA) was performed in N3 and K3 GSCs transfected with miR-NC, miR-129-5p or miR-129-5p plus Wnt5a (Left panel). Representative images of neurosphere formation capacity in the presence of miR-129-5p overexpression were captured following the neurosphere formation assay (Right panel) (six replicates per group, three independent experiments per group). Scale bar, 100 μm. **d** Proliferation of miR-NC, miR-129-5p or miR-129-5p plus Wnt5a-transfected N3 and K3 GSCs upon TMZ treatment (300 μM) was tested every 24 h (six replicates per group, three independent experiments per group). **e** Cell proliferation was detected in GBM cells stably expressing miR-NC or miR-129-5p, without or with TMZ treatment at different doses (six replicates per group, three independent experiments per group). The CCK-8 assay was performed 48 h after treatment. **f** Proliferation of miR-NC, miR-129-5p or miR-129-5p plus Wnt5a-transfected U251 and N3 cells upon TMZ treatment (200 μM) was tested every 24 h (six replicates per group, three independent experiments per group). **g** Representative images and quantification of EdU analysis of miR-NC, miR-129-5p or miR-129-5p plus Wnt5a-transfected U251 and N3 cells treated with TMZ (200 μM) for 48 h (six replicates per group, three independent experiments per group). Scale bar, 100 μm. **h** Western blot analysis was used to detect regulation of the apoptosis-related proteins cleaved caspase 3, cleaved caspase 7, Bcl-2 and Bax by miR-129-5p in U251 or N3 cells upon TMZ treatment (200 μM) for 48 h (three replicates per group, three independent experiments per group). Data are expressed as the mean ± s.e.m

We enriched N3 and K3 neurospheres by culturing them in serum-free stem cell medium^24^. After 7–10 days, N3 and K3 neurospheres were visible in culture under the microscope (Fig. 5a). Immunofluorescence staining indicated that the GSC markers, CD133 and Nestin^26,27^, were expressed in N3 and K3 neurospheres (Fig. 5a). We overexpressed miR-129-5p mimics in N3 and K3 cells (N3/miR-129-5p and K3/miR-129-5p) (Fig. 3a). N3/miR-129-5p and K3/miR-129-5p neurospheres showed markedly decreased stem cell marker expression (Sox2, Oct4) and increased astrocytic marker (GFAP) relative to control neurospheres (Fig. 5a, b). Meanwhile, miR-129-5p overexpression in differentiating N3 and K3 GSC cultures attenuated expression of Sox2 and Oct4 and increased GFAP expression, confirming that in differentiating conditions, miR-129-5p upregulation promoted astrocytic differentiation. Forced expression of Wnt5a partially abolished miR-129-5p-mediated inhibition of GSC stemness (Supplementary Fig. 2A-C). By performing limiting dilution assays, we demonstrated that overexpression of miR-129-5p inhibited neurosphere formation of N3 and K3 cells (Fig. 5c). Enforced Wnt5a expression restored the expression of stem cell markers and neurosphere formation (Fig. 5a–c). To determine the role of miR-129-5p in chemotherapy, we exposed N3 and K3 GSCs overexpressing miR-129-5p or miR-NC to different concentrations of TMZ and demonstrated that the IC50 for GSCs was ~300 μM (data not shown). miR-129-5p-overexpressing GSCs showed markedly lower viability than control GSCs upon exposure to TMZ (300 μM) (Fig. 5d). miR-129-5p-overexpressing U251 and N3 monolayer cells also showed decreased cell viability upon 48 h TMZ treatment compared to that in control cells (Fig. 5e). We then examined cell viability in the presence of TMZ (200 μM) at different time points. Overexpression of miR-129-5p significantly decreased cell survival of U251 and N3 cells in the presence of TMZ (200 μM) (Fig. 5f). Forced expression of Wnt5a reversed the miR-129-5p-mediated chemosensitivity (Fig. 5d–f).

EdU assays revealed that miR-129-5p-overexpressing cells exhibited decreased EdU-positive cells, whereas control GBM cells presented a higher number of EdU-positive cells in the presence of TMZ (200 μM) for 48 h (Fig. 5g). Overexpression of Wnt5a restored the proliferation function in TMZ-treated cells (Fig. 5g). We compared the expression levels of apoptosis-related proteins between miR-NC- and miR-129-5p-transfected cells. miR-129-5p overexpression significantly increased the protein levels of cleaved caspase 3, cleaved caspase 7 and Bax, while decreasing the expression levels of the apoptosis inhibitor Bcl-2 in U251 and N3 cells upon TMZ treatment (200 μM) for 48 h (Fig. 5h). Here, our data suggested that miR-129-5p inhibits neurosphere formation and confers TMZ chemosensitivity on GBM cells by targeting Wnt5a.

### Downregulation of miR-129-5p expression activates the PKC/ERK/NF-κB and JNK pathways by targeting Wnt5a

**Fig. 6:**
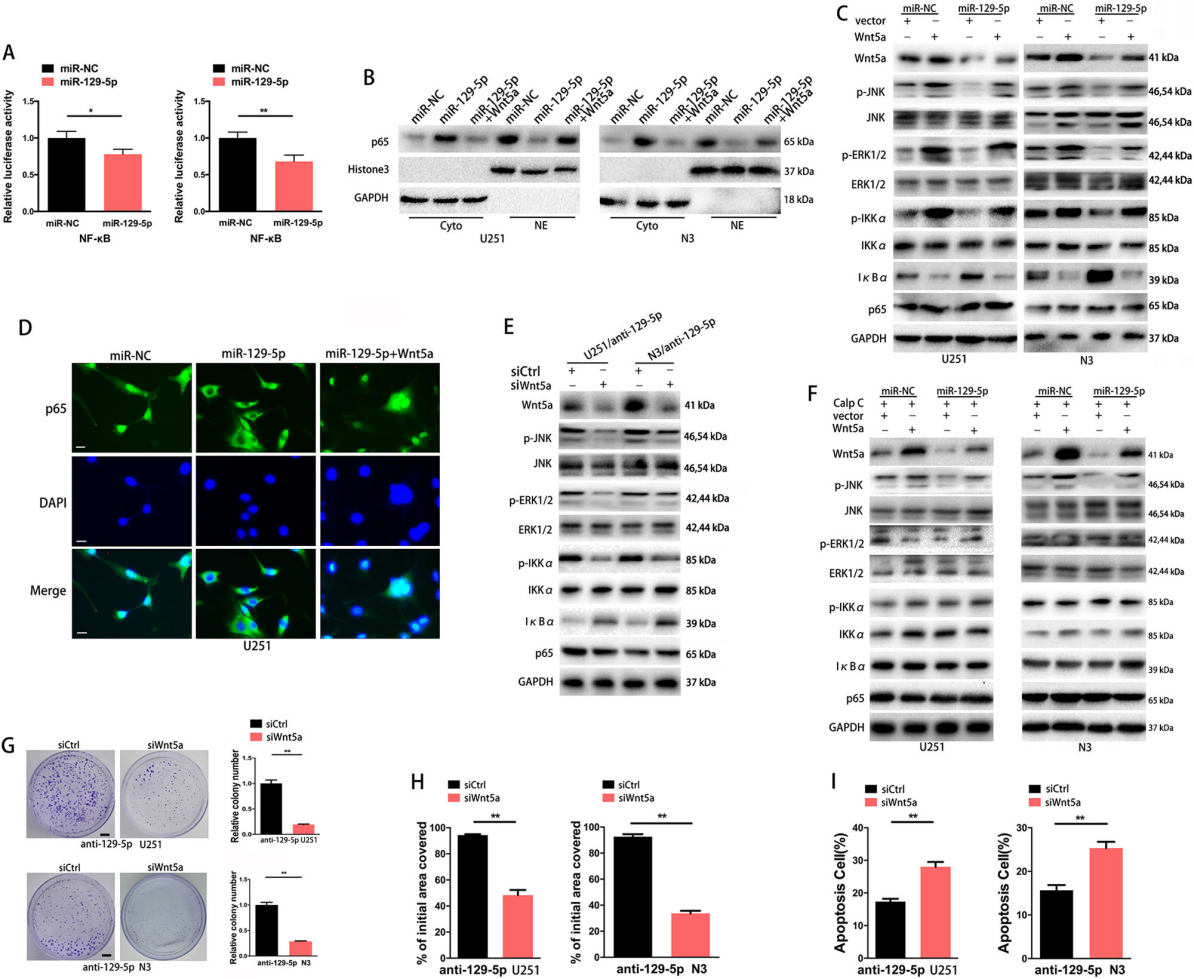
Downregulation of miR-129-5p expression activates PKC/ERK/NF-κB and JNK pathways by targeting Wnt5a. **a** Luciferase assay of the interaction between NF-κB signalling and miR-129-5p in GBM cells (six replicates per group, three independent experiments per group). **b** Western blot analysis of p65, GAPDH in cytoplasmic extracts (Cyto) and p65, Histone3 in nuclear extracts (NE) in miR-NC, miR-129-5p or miR-129-5p plus Wnt5a-transfected U251 and N3 cells (three replicates per group, three independent experiments per group). **c** Western blot analysis of p-JNK, total JNK, p-ERK1/2, total ERK1/2, p-IKKα, IKKα, IҡBα and p65 in U251/miR-NC, U251/miR-129-5p, N3/miR-NC and N3/miR-129-5p cells co-transfected with the vector or Wnt5a (three replicates per group, three independent experiments per group). GAPDH was used as the loading control. **d** Immunofluorescence assay was conducted on GBM cells overexpressing miR-NC or the miR-129-5p mimic. miR-129-5p blocked the accumulation of p65 in the nuclei of U251 GBM cells. Overexpression of Wnt5a rescued the accumulation of p65 in the nucleus attenuated by miR-129-5p (three replicates per group, three independent experiments per group). **e** Western blot analysis of p-JNK, total JNK, p-ERK1/2, total ERK1/2 and NF-κB activation-associated proteins in siCtrl or siWnt5a U251 or N3 cells with miR-129-5p inhibition (three replicates per group, three independent experiments per group). **f** U251 or N3 cells overexpressing miR-NC or miR-129-5p were incubated with the PKC inhibitor, Calp C (100 nM). Western blot analysis revealed that Wnt5a-mediated activation of ERK and NF-κB, but not JNK signalling, was blocked when PKC was neutralised (Calp C) (three replicates per group, three independent experiments per group). **g** Enforced deletion of Wnt5a dampened colony formation in U251 or N3 GBM cells with miR-129-5p inhibition (six replicates per group, three independent experiments per group). **h** Enforced deletion of Wnt5a dampened the invasion in U251 or N3 GBM cells with miR-129-5p inhibition (six replicates per group, three independent experiments per group). **i** Enforced deletion of Wnt5a dampened the anti-apoptotic effect of miR-129-5p inhibition in U251 or N3 GBM cells upon TMZ treatment (200 μM) for 48 h (six replicates per group, three independent experiments per group). Data are expressed as the mean ± s.e.m

NF-κB has been demonstrated to be constitutively activated and acts as a key factor in the development and progression of various cancers^28,29^. Wnt5a has been demonstrated to activate NF-κB pathway-dependent survival signalling in chronic lymphocytic leukaemia cells^13,30^. Here, we investigated whether the NF-κB pathway was involved in miR-129-5p/Wnt5a signalling in GBM cells. miR-129-5p overexpression reduced the luciferase activity of the NF-κB reporter, indicating that miR-129-5p contributed to NF-κB inactivation (Fig. 6a). Surprisingly, the expression of p65—a key member of the NF-κB family—did not change in U251/miR-129-5p cells compared to that in control cells (Fig. 6c). However, p65 expression levels were elevated in cytoplasmic extracts and reduced in nuclear extracts of U251/miR-129-5p cells compared to those in U251/miR-NC cells (Fig. 6b). To further investigate the role of miR-129-5p in the NF-κB activation process, we assessed the phosphorylation levels of IKKα and IκBα expression in miR-129-5p-overexpressing GBM cells (Fig. 6c). miR-129-5p overexpression led to reduced phosphorylation of IKKα and elevated IκBα expression. Overexpression of Wnt5a rescued NF-κB activation and the accumulation of p65 in the nucleus that was inhibited by miR-129-5p (Fig. 6b, c). miR-129-5p attenuated the accumulation of p65 in the nucleus in a Wnt5a-dependent manner (Fig. 6d). Parallel experiments were performed in miR-129-5p-overexpressing N3 cells and similar results were observed (Supplementary Fig. 3A).

The results presented in Figs. 3–5 suggested that miR-129-5p expression contributed to a variety of malignant phenotypic changes. Thus, we hypothesised that low miR-129-5p expression activates multiple Wnt5a-associated signalling pathways that are involved in GBM progression. Therefore, we determined the status of the ERK and JNK oncogenic signalling pathways in GBM cells. Cellular levels of p-ERK1/2 and p-JNK were markedly decreased in U251 and N3 cells stably overexpressing miR-129-5p compared to those in control cells overexpressing miR-NC, while no statistically significant reduction of total ERK1/2 and JNK was observed (Fig. 6c). Enforced Wnt5a expression restored the ERK and JNK signalling pathways that were inhibited by miR-129-5p overexpression. A pool of three Wnt5a-specific siRNAs (siWnt5a) or a control (siCtrl) counterpart were transfected into U251 and N3 cells with miR-129-5p inhibition (U251/anti-129-5p and N3/anti-129-5p) (Supplementary Fig. 3B; U251: 0.20 ± 0.01-fold relative expression; N3: 0.12 ± 0.02-fold relative expression). Forced deletion of Wnt5a abrogated ERK and JNK signalling activation and the accumulation of p65 in the nucleus of GBM cells in U251/anti-129-5p and N3/anti-129-5p (Fig. 6e). PKC–the upstream modulator of ERK and NF-κB–is involved in the Wnt5a-stimulated NF-κB pathway in multiple types of cancers^31–34^. When GBM cells were pre-incubated with the PKC inhibitor, Calphostin C (Calp C) immunoblotting revealed that the Wnt5a-mediated activation of ERK and NF-κB, but not JNK signalling, was blocked when PKC was neutralised (Fig. 6f). Taken together these results indicate that downregulation of miR-129-5p activates the Wnt5a-stimulated PKC/ERK/NF-κB and JNK pathways in GBM cells.

U251/anti-129-5p and N3/anti-129-5p cells with a deletion of Wnt5a displayed significantly inhibited colony formation and invasion and a lower rate of apoptosis upon TMZ treatment compared to the levels observed in U251/anti-129-5p and N3/anti-129-5p cells transfected with siCtrl (Fig. 6g–i). Altogether these results indicate that inhibition of Wnt5a in GBM cells with low miR-129-5p expression reverses malignant phenotypes in GBM.

### miR-129-5p inhibits tumour growth in vivo and elevated Wnt5a is associated with decreased survival

**Fig. 7:**
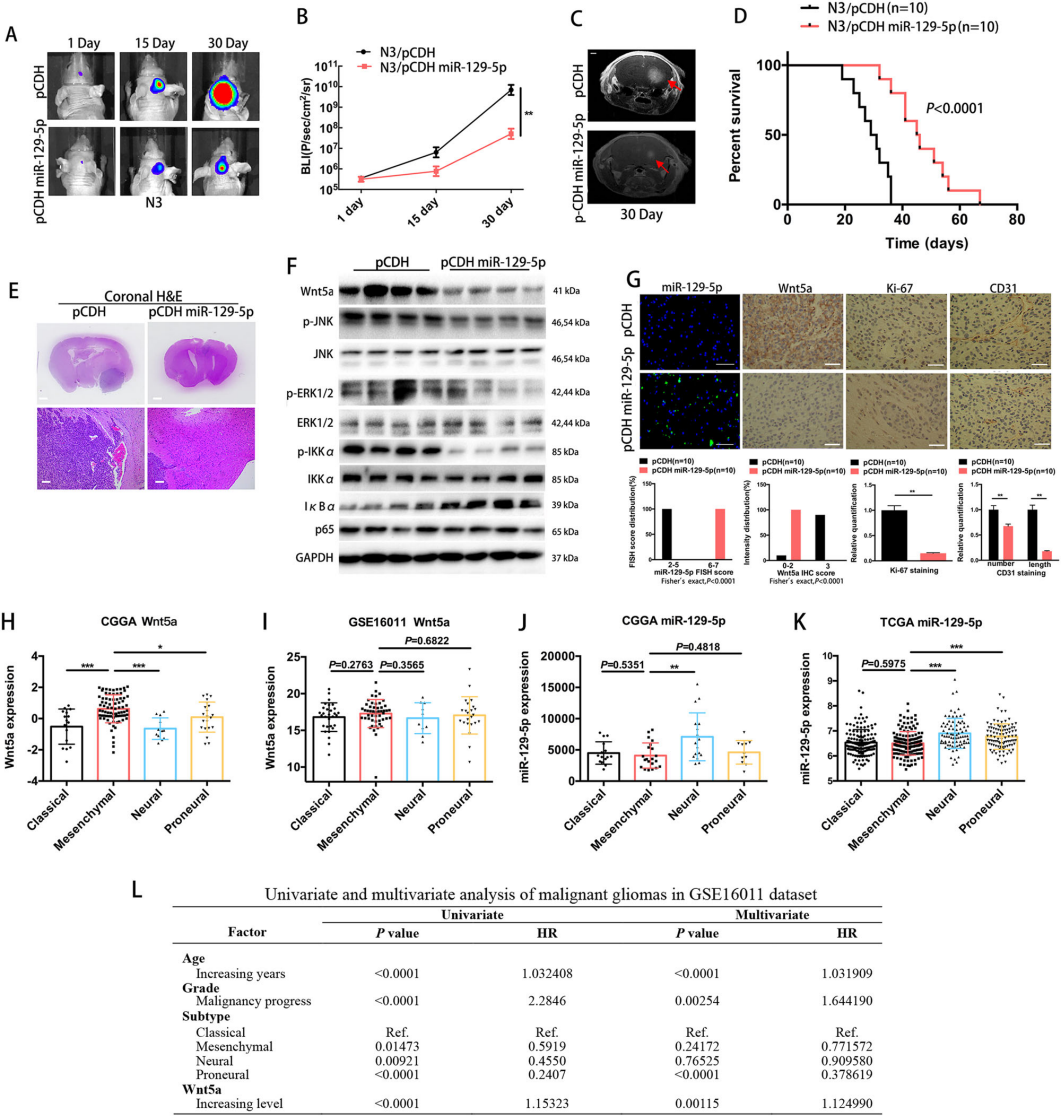
miR-129-5p inhibits tumour growth and angiogenesis in vivo by targeting Wnt5a . **a** Representative bioluminescence images of mice bearing intracranial pCDH- or pCDH miR-129-5p-transfected N3 cells on the days indicated (*n* = 10 each group). **b** Bioluminescence was quantified in tumours from 2 groups. **c** Representative images of T2-weighted MRI of intracranial tumour growth (arrows) at day 30 in mice bearing pCDH- or pCDH miR-129-5p-transfected N3 cells. Scale bar, 1 mm. **d** Kaplan–Meier survival curve of mice injected with pCDH- or pCDH miR-129-5p-transfected N3 cells (Log-rank test*, P* < 0.0001). **e** Representative haematoxylin and eosin (H&E) staining for tumour cytostructure. Scale bar, 1 mm (upper) and 100 μm (lower). **f** Expression levels of p-JNK, total JNK, p-ERK1/2, total ERK1/2 and NF-κB activation-associated proteins in xenografts from mice bearing pCDH- or pCDH miR-129-5p-transfected N3 cells (three replicates per group, three independent experiments per group). **g** FISH analysis of miR-129-5p and immunohistochemical analysis of paired sections of Wnt5a, Ki-67 and CD31 expression in intracranial tumours originating from pCDH- or pCDH miR-129-5p-transfected N3 cells (three replicates per group, three independent experiments per group). Scale bar, 50 μm. **h**–**i** Wnt5a expression in molecular GBM subtypes in the CGGA and GSE16011 data sets. **j**–**k** miR-129-5p expression in molecular GBM subtypes in the CGGA and TCGA data sets. **l** Univariate and multivariate analysis of malignant gliomas in the GSE16011 data set

We established an orthotopic GBM model to analyse the in vivo function of miR-129-5p. Representative images of mice implanted with intracranial tumours are shown in Fig. 7a. Bioluminescence imaging demonstrated a statistically significant difference in tumour volume between two groups implanted with N3 cells transfected by the lentiviral control pCDH or pCDH miR-129-5p, confirmed by RT-qRCR (Supplementary Fig. 4A and Fig. 7a, b). MRI analysis also confirmed that overexpression of miR-129-5p in N3 led to a decrease in xenograft volume (Fig. 7c). The median survival of mice bearing intracranial pCDH-transfected tumour xenografts was 30 days, whereas mice bearing the pCDH miR-129-5p-transfected xenografts had a median survival of 46 days (Fig. 7d; log-rank test, *P* < 0.0001). Haematoxylin and eosin (H&E) staining confirmed this finding that mice bearing N3 cells transfected with pCDH miR-129-5p showed a drastic decrease in tumour volume (Fig. 7e). Moreover, important pathway proteins, such as p-JNK, p-ERK1/2 and NF-κB activation-associated proteins, were significantly inhibited by miR-129-5p overexpression in GBM xenografts (Fig. 7f). Immunohistochemistry analysis also revealed that tumours derived from miR-129-5p-overexpressing N3 showed increased miR-129-5p, decreased Wnt5a expression (Fig. 7g, left), a lower proliferation index as demonstrated by Ki-67 staining and significantly lower levels of CD31 expression (Fig. 7g, right). Altogether these results indicate that miR-129-5p overexpression inhibits tumour growth and angiogenesis in vivo through the downregulation of Wnt5a.

We determined the expression levels of miR-129-5p and Wnt5a in 4 molecular GBM subtypes^35^. Consistent with the finding^19^ that the highest Wnt5a expression was observed in the mesenchymal subtype in the TCGA data set, mesenchymal GBMs significantly expressed the highest levels of Wnt5a in the CGGA data set (Fig. 7h). Furthermore, in the GSE16011 data set, mesenchymal GBMs tended to express high levels of Wnt5a, although it did not reach statistical significance (Fig. 7i). Conversely, mesenchymal GBMs tended to express the lowest miR-129-5p levels in both the TCGA and CGGA data sets (Fig. 7j, k), although this result did not reach statistical significance. These findings indicated that high Wnt5a expression may be associated with the highly infiltrating^19^ and aggressive phenotypic^36^ characteristics of mesenchymal GBM cells.

Wnt5a is an independent predictor of poor prognosis in GBM^19,20^, which accounts for ~40% of glioma. To fully explore the association of Wnt5a expression with survival in all malignant gliomas, we conducted univariate and multivariate Cox regression analysis to assess the prognostic independence of Wnt5a expression among other factors. Increasing Wnt5a expression was correlated with a worse outcome in malignant glioma patients in all included data sets (Fig. 7l and Supplementary Table 1 and 2). Increasing Wnt5a was independently correlated with a worse outcome in malignant glioma patients in the GSE16011 data set (Fig. 7l). However, in the Rembrandt and CGGA data sets, Wnt5a expression was a prognosis predictor dependent on other factors (Supplementary Table 1 and 2). These results indicated that Wnt5a expression may be an independent prognostic factor in malignant glioma; however, the results need to be validated using a larger data set.

## Discussion

Our findings demonstrate that miR-129-5p acts as a tumour suppressor, mediating the inhibition of proliferation, invasion, migration, neurosphere formation, angiogenesis and TMZ resistance by suppressing its target, Wnt5a.

Wnt5a plays an important role in tumour-progressive functions^12,13,37^. Ror1/2 and Ryk act as receptors or coreceptors for Wnt5a, and Ror1/2 and Ryk expression positively correlates with Wnt5a expression in GBM ^12,13,15^. Wnt5a-Ror2 interaction activated the signalling cascades involving JNK, such as the PCP pathway^13,15^. Here, we demonstrate that Wnt5a is targeted by miR-129-5p, whose expression is inversely correlated with Wnt5a in glioma. miR-129-5p directly binds to a site on the Wnt5a-3′ UTR, which leads to the suppression of the Wnt5a downstream pathway in vitro and in vivo.

Blocked NF-κB activity reduced cyclin E1 and cyclin D1 expression, which regulates cell cycles in various cancers through cell-cycle checkpoints^38,39^, thus inhibiting the passage through the restriction point in late G1^38^. We observed that miR-129-5p-mediated downregulation of Wnt5a inactivates the NF-κB pathway in GBM cells and blocks cell-cycle progression. The formation of abnormal tumour vasculature is thought to be one of the major obstacles to the treatment of GBM^40^. miR-129-5p inhibits Wnt5a and VEGF secretion, both of which act as pro-angiogenic modulators in GBM tumourigenesis^41^. We also observed that miR-129-5p inhibited invasion and migration of GBM cells by targeting Wnt5a. EMT has emerged as a key regulator facilitating GBM cell invasion and migration^42^. miR-129-5p overexpression inhibits EMT signalling pathways in GBM cells by targeting Wnt5a. To create a path for invasion, GBM cells must be able to pass the physical barrier, extracellular matrix (ECM). Inhibited Wnt5a expression also blocks MMP9 and MMP2 expression, which are involved in the degradation of ECM proteins. Cancer stem cells are considered as mediators of chemoresistance^43,44^. miR-129-5p overexpression blocks GBM stem cell markers, inhibits the capability of neurosphere formation and elevates chemosensitivity of GBM cells to TMZ therapy.

In summary, miR-129-5p downregulation plays an important role in the progression and malignancy of GBM. miR-129-5p is a critical repressor of Wnt5a. Understanding the dysregulation of miR-129-5p/Wnt5a-stimulated PKC/ERK/NF-κB and JNK pathways in GBM is important to improve our knowledge of the biological basis of GBM development and progression and has therapeutic potential in the treatment of GBM.

## Materials and Methods

### Patients and samples

Human glioma samples and normal brain tissues from the Department of Neurosurgery at the First Affiliated Hospital of Nanjing Medical University were included in this study. This study protocol was approved by the hospital institutional review board and written informed consent was obtained from each participant. Tissue samples, including 6 normal brain, 8 WHO grade II, 8 grade III and 8 grade IV (GBM) tissues were obtained from the routine therapeutic surgery of patients treated between April 2011 and September 2016. Patients were enroled in the study if their diagnosis was confirmed histologically by two neuropathologists based on the 2007 WHO classification guidelines. Glioma cases from the Chinese Glioma Genome Atlas (CGGA, http://www.cgga.org.cn) were included in this study.

### Cell culture and reagents

U251, LN229, A172, LN18 and T98G cells were incubated in Dulbecco’s Modified Eagle’s medium (DMEM) supplemented with 10% foetal bovine serum (FBS), purchased from the American Type Culture Collection (ATCC). Primary human N3 and K3 GBM cells were obtained as follows: Two primary human GBM samples were washed, acutely dissociated in oxygenated artificial cerebrospinal fluid and subjected to enzymatic dissociation, as described previously^45^. HBMVECs (Sciencell) and neurospheres were incubated as described previously^24^. NHAs were grown in the astrocyte growth media supplemented with rhEGF, insulin, L-glutamine, GA-1000, ascorbic acid and 5% FBS, purchased from Lonza. To assure the authenticity of the cell lines, we prepared frozen stocks from initial stocks and used a new frozen stock for the experiments every 3 months. To induce GSC differentiation, GSCs were dissociated and cultured on polyornithine and fibronectin double-coated plates in differentiating conditions (Neurobasal media supplemented with N2, B27, 3 mM L-glutamine, and 5% FBS)^46^. Sources and concentrations of the reagents used were 100 nM Calp C (Merck, Whitehouse Station, NJ, USA) and 25 to 400 μM TMZ (Sigma-Aldrich, St. Louis, MO, USA).

### Transfection and stable cell line establishment

All transfections were conducted using Lipofectamine 2000 (Invitrogen, Carlsbad, CA, USA) according to the manufacturer’s instructions. miR-129-5p mimic, miR-129-5p inhibitor and their related negative controls (RiboBio Co. Ltd, Guangzhou, China) were transfected into cells at a concentration of 40 nM, and the cells were maintained for 48 h after transfection. Constructs containing siWnt5a and its negative control siRNA (siCtrl), and pcDNA3.1-Wnt5a (not including the 3′ UTR) and its control pcDNA3.1 vector were used. A lentiviral pCDH empty vector (pCDH) and miR-129-5p expressing vector (pCDH miR-129-5p) were purchased from GeneChem Co. Ltd. (Shanghai, China).

### RNA isolation and real-time quantitative PCR

TRIzol reagent was used to extract total RNA from cells or human tissues according to the manufacturer’s instructions. To quantify the expression levels of miR-129-5p, the stem-loop-specific primer method was used^47^. miRNA-specific reverse transcription primers and quantitative PCR primers were obtained from RiboBio Co. Ltd. (Guangzhou, China). U6 levels were used as the internal control. To analyse the mRNA levels of Wnt5a, total RNAs were reverse transcribed by oligodT primers using the PrimeScript RT Reagent kit (Takara, Dalian, China). GAPDH served as an internal control. Primers used in quantitative PCR analysis were as follows: Wnt5a: forward, 5′-ATTCTTGGTGGTCGCTAGGTA-3′; reverse: 5′-CGCCTTCTCCGATGTACTGC-3′. RT-qPCR was conducted using the SYBR Premix DimerEraser on a 7900HT system and fold changes were calculated by relative quantification (2^−△△Ct^).

### Western blot and ELISA

Protein extraction and western blotting were conducted as described previously^47–49^. Antibodies against mammalian vimentin, slug, E-cadherin, N-cadherin, MMP2, IKKα, total JNK, p-JNK, p-ERK1/2, total ERK1/2, cyclin E1, cyclin D1, cleaved caspase 3 and cleaved caspase 7 were purchased from Cell Signalling Technology. The antibody against GAPDH was obtained from Bioworld. Antibodies against IκBα, p-IKKα (Thr23) and p65 were from Santa Cruz Biotechnology. Antibodies against Wnt5a, MMP9, Oct4, Sox2, Bcl-2, Bax, β-actin (Abcam), CD133 (Amersham) and Nestin (BD) were also used. Concentrations of secreted Wnt5a and VEGF were determined using the Human Protein Wnt-5a (WNT5A) ELISA kit and Human Vascular Endothelial Cell Growth Factor (VEGF) ELISA kit (Cusabio, Wuhan, China).

### Luciferase assays

The 3′-UTR of Wnt5a was synthesised and annealed, then inserted into the SacI and HindIII sites of the pMIR-reporter luciferase vector (Ambion) downstream of the stop codon of the gene for luciferase. The sequences complementary to the binding site of miR-129-5p in the 3′-UTR (Wnt5a: GCAAAAA) were replaced by TACCCCC for mutagenesis of the binding site. These constructs were validated by sequencing. U251 and N3 cells were seeded in triplicate in 24-well plates and cultured for 24 h. The cells were co-transfected with the wild-type or mutated plasmid and indicated amounts of miR-129-5p or miR-NC mimics. A Dual Luciferase Reporter Assay kit (Promega) was used to conduct luciferase assays 24 h after transfection according to the manufacturer’s instructions.

### Cell proliferation

CCK-8 and plate colony formation assays were performed to evaluate the proliferative ability of the cells^22,24^. The cells were seeded in 24-well plates overnight and then transfected with miR-NC, miR-129-5p or miR-129-5p plus pcDNA3.1-Wnt5a. GBM stem cells or monolayer cells were trypsinized and seeded in 96-well plates at a confluence of 2000 cells per well per 100 μL of stem cells or 10% FBS supplemented DMEM. Absorption of the cells was measured at different indicated time points using the CCK8 kit (Dojindo Laboratories, Kumamoto, Japan) following the manufacturer’s instructions. An EdU imaging kit (Life Technologies) was used to determine DNA synthesis of cells grown on coverslips in a 24-well dish after appropriate TMZ treatments. Immunostaining and EdU assay results were visualised using a Leica DMI3000B microscope. EdU-positive cells were manually counted.

### Immunofluorescent staining

For immunostaining analysis of undifferentiated neurospheres, the cells were fixed with 4% paraformaldehyde, washed with PBS and incubated with primary antibodies against Nestin (Abcam) and CD133/1 (Miltenyi Biotec). Appropriate secondary antibodies (FITC Green goat anti-mouse and Cy3 Red goat anti-rabbit; Molecular Probes, USA) were used and the cell nucleus was stained with DAPI. For immunostaining analysis of NF-κB pathway activity, briefly, cells were fixed with 4% formaldehyde, washed with PBS twice, blocked with 5% normal goat serum in PBS for 1 h and then incubated with a primary antibody against p65 overnight at 4 °C. Following this, the cells were washed, incubated with secondary antibodies and then stained with DAPI before visualisation using fluorescence microscopy (Leica DMI3000B, Germany).

### In vitro 3D migration assays and other assays

For wound-healing assays, cell layers were scratched using a 20 μL tip to make wounded gaps once they reached a confluence of 90% in 6-well plates, then washed with PBS twice and observed at indicated times. For 3D migration assays, a drop (20 μL) of the cell suspension (containing 1000 cells) was placed onto the lid of a 10-cm dish^50^. The lids were then inverted over dishes containing 10 ml PBS. Following a 2-day culture of hanging drops, the resulting cellular aggregates were harvested and implanted into three-dimensional collagen I gels^50^. Collagen I gels (PureCol, Inamed, Fremont, CA, USA) were prepared by adjusting the pH to 7.5 using 1 N NaOH and supplemented with 2% FBS and DMEM. After polymerisation at 37 °C, the collagen gel was overlaid with 300 μL of DMEM supplemented with 10% FBS. The results were monitored using a Leica DMI3000B microscope system. For vessel-forming of HBMVECs, growth factor-reduced Matrigel (200 μL) (BD Biosciences) was thawed on ice, placed onto each well of an 8-chamber polystyrene vessel tissue culture-treated glass slides, and allowed to gelatinise for half hour in 37 °C incubator. A total of 5 × 10^4^ HBMVECs were then seeded into each well of the culture slides in MCDB-131 medium. After 16 h, tube formation was monitored by Leica DMI3000B phase contrast microscopy. For neurosphere formation assays, neurosphere cells were dissociated into single cells. The cells with decreasing numbers (100, 50, 25, 12) per well, plated in 96-well plates containing stem cell medium, were used^51^. Extreme limiting dilution analysis was conducted using the software available at http://bioinf.wehi.edu.au/software/elda/. Flow cytometry-based cell-cycle analysis was performed using FACScan (Beckman Gallios) with the ModFit software and displayed as a percentage of cells in a particular phase.

### MRI of orthotopic mouse tumours

Intracranial tumour growth was analysed in vivo in isoflurane-anaesthetised mice after inoculation. A Bruker 7.0 T scanner (Bruker BioSpin GmbH) was used to visualise the intracranial xenografts^22^. T2-weighted images were obtained by a rapid acquisition relaxation-enhanced sequence using the following parameters: relaxation time (4000 milliseconds), echo time (15 milliseconds), scan time (200 s), view field (25.6 × 25.6 mm), slice thickness (0.8 mm) and matrix (256 × 256).

### Isolation of RISC-associated RNA

N3 and U251 cells overexpressing miR-129-5p or miR-NC were fixed with 1% formaldehyde, followed by chromatin fragmentation. For immunoprecipitation, cells were lysed in NETN buffer and then incubated with Dynabeads Protein A (Invitrogen) supplemented with IgG control or anti-Pan-Ago, clone 2A8 antibody (Millipore). The immunoprecipitated RNA was released by proteinase K digestion and extracted using phenol/chloroform/isopropyl alcohol. RNA was purified by ethanol precipitation with glycogen, resolved, and treated with DNase I.

### Fluorescence in situ hybridisation

miR-129-5p expression in human glioma samples and nude mouse xenografts was determined using FISH, as described previously^22^. The number of positively stained cells was jointly scored without knowledge of clinical information on a scale of 1–4, with 1 = ≤ 10% of cells positively stained, 2 = 10–30% of cells positively stained, 3 = 30–70% of cells positively stained and 4 = ≥ 70% of cells positively stained. The staining intensity was evaluated on a scale of 1–3, with 1 = low intensity, 2 = medium intensity and 3 = high intensity. The scores of 2 items together were divided into 3 groups for quantitative analysis of miR-129-5p expression: 2–3 = negative(loss), 4–5 = positive and 6–7 = strong positive.

### Immunohistochemistry (IHC) and Haematoxylin and eosin (H&E) stain

To detect Wnt5a, Ki-67 or CD31 (Abcam) expression, IHC was performed on tumour tissue from nude mouse xenografts using methods described previously^22,24^. Coronal H&E staining of tumour tissue from nude mouse xenografts was performed as described previously^22^. Ten visual fields from different areas of every tumour were employed for staining evaluation. Sections stained with Wnt5a were scored by an IHC score based on staining intensity and percentage of positive cells within the whole tissue section^52^. The IHC score was calculated by multiplying the staining intensity with the corresponding positive cells (1 for low, 2 for moderate and 3 for high).

### Xenograft tumour assay

Male BALB/c nude mice (6 weeks old) were used. All described procedures involving experimental animals were performed in agreement with standard guidelines under a protocol approved by Nanjing Medical University. For establishing intracranial GBM, 5.0 × 10^4^ N3 cells stably expressing the luciferase reporter were stereotactically implanted^24^. Mice were analysed for Fluc activity by bioluminescence imaging. Before imaging, each mouse underwent an intraperitoneal injection of D-luciferin (10 μl g^−1^).

### Statistical analysis

Data are expressed as the mean ± s.e.m of three independent experiments. Student’s *t*-test was used to evaluate statistical significance, and the survival analysis was performed using log-rank tests. Multiple group FISH scores were compared using the Chi-square test. The variance was similar between the compared groups. Univariate Cox and backward stepwise multivariate Cox regression analyses were performed with the survival package^53^ of R 3.3.3, taking vital clinical status and molecular subtypes into account. *P*-values < 0.05 (two-sided) were considered significant (single asterisks in the figures). *P*-values < 0.01 (two-sided) were strongly significant (double asterisks).

## Electronic supplementary material

Supplementary materials and methods(DOCX 3868 kb)
