# Supplementary material for: miR-129-5p targets Wnt5a to block PKC/ERK/NF-κB and JNK pathways in glioblastoma
Source: Cell Death Dis. 2018 Mar 12;9(3):394. doi: 10.1038/s41419-018-0343-1 (PMC5847604; doi:10.1038/s41419-018-0343-1)
Supplement: Supplementary file 1 — Supplementary materials and methods(DOCX 3868 kb) [file 41419_2018_343_MOESM1_ESM.docx]

**Supplementary Figure Legends**

**Supplementary Figure 1 (Related to Figure 2).**

A, qRT-PCR analysis of miR-129-5p in N3 and U251 cells transfected with miR-129-5p mimics, or their related negative control(miR-NC mimics). U6 RNA served as the loading control.

B, Expression of Wnt5a was analyzed in NHAs and eight GBM cells (six replicates per group, three independent experiments per group). **(*P*<0.01) and ***(*P*<0.001) indicate signiﬁcant difference compared with NHAs. Data are expressed as the mean ± s.e.m.


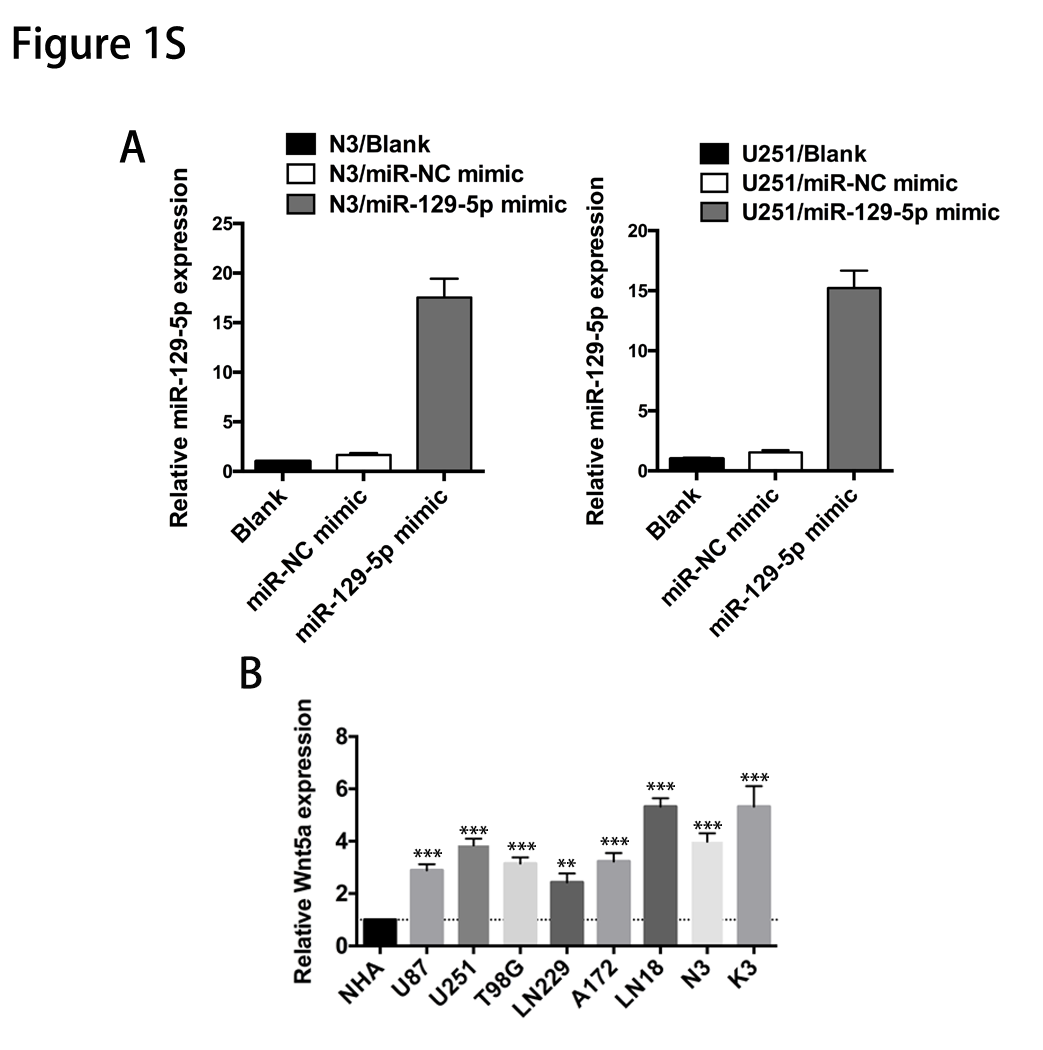


**Supplementary Figure 2 (Related to Figure 5).**

A-C, The effect of miR-129-5p overexpression on stemness and differentiation of GSCs. N3 and K3 GSCs were plated in differentiation conditions as described and transfected with miR-NC or miR-129-5p mimics. The expression of stemness (Sox2, Oct4) and astrocytic marker (GFAP) were detected by RT-qPCR analysis(three replicates per group, three independent experiments per group). *P*< 0.05 indicated by one asterisk, *P*< 0.01 by two asterisks, and *P*< 0.001 by three asterisks.


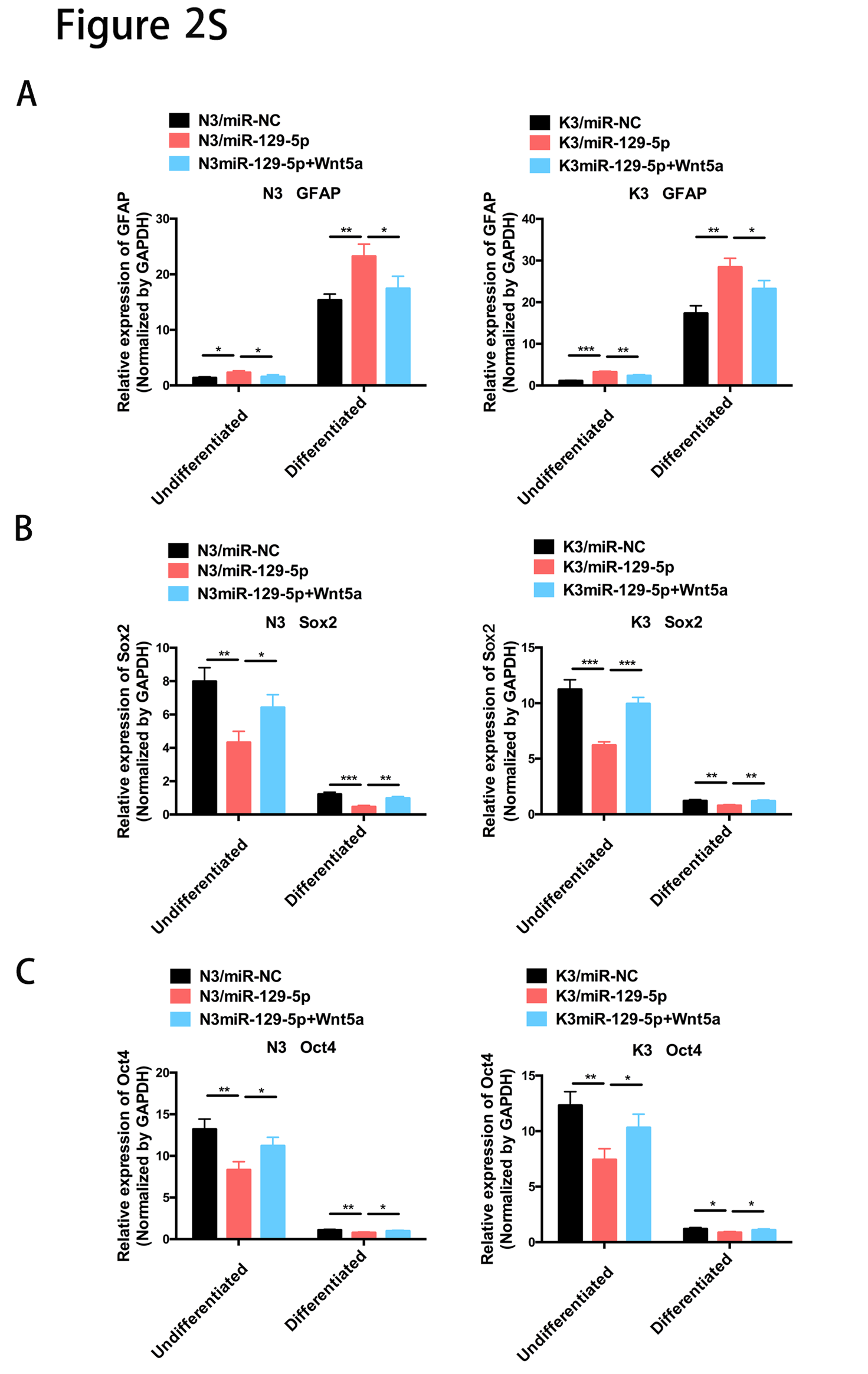


**Supplementary Figure 3 (Related to Figure 6).**

A, Immunofluoresence assay was conducted on N3 cells transfected with miR-NC or miR-129-5p. MiR-129-5p blocked the accumulation of p65 in nuclei of N3 cells. Overexpression of Wnt5a rescued the accumulation of p65 in nuclei that was attenuated by miR-129-5p (three replicates per group, three independent experiments per group).

B,U251/anti-129-5p or N3/anti-129-5p cells were transfected with a pool of Wnt5a-specific siRNA (siWnt5a) or control (siCtrl), followed by RT-qPCR analysis.GAPDH RNA served as the loading control


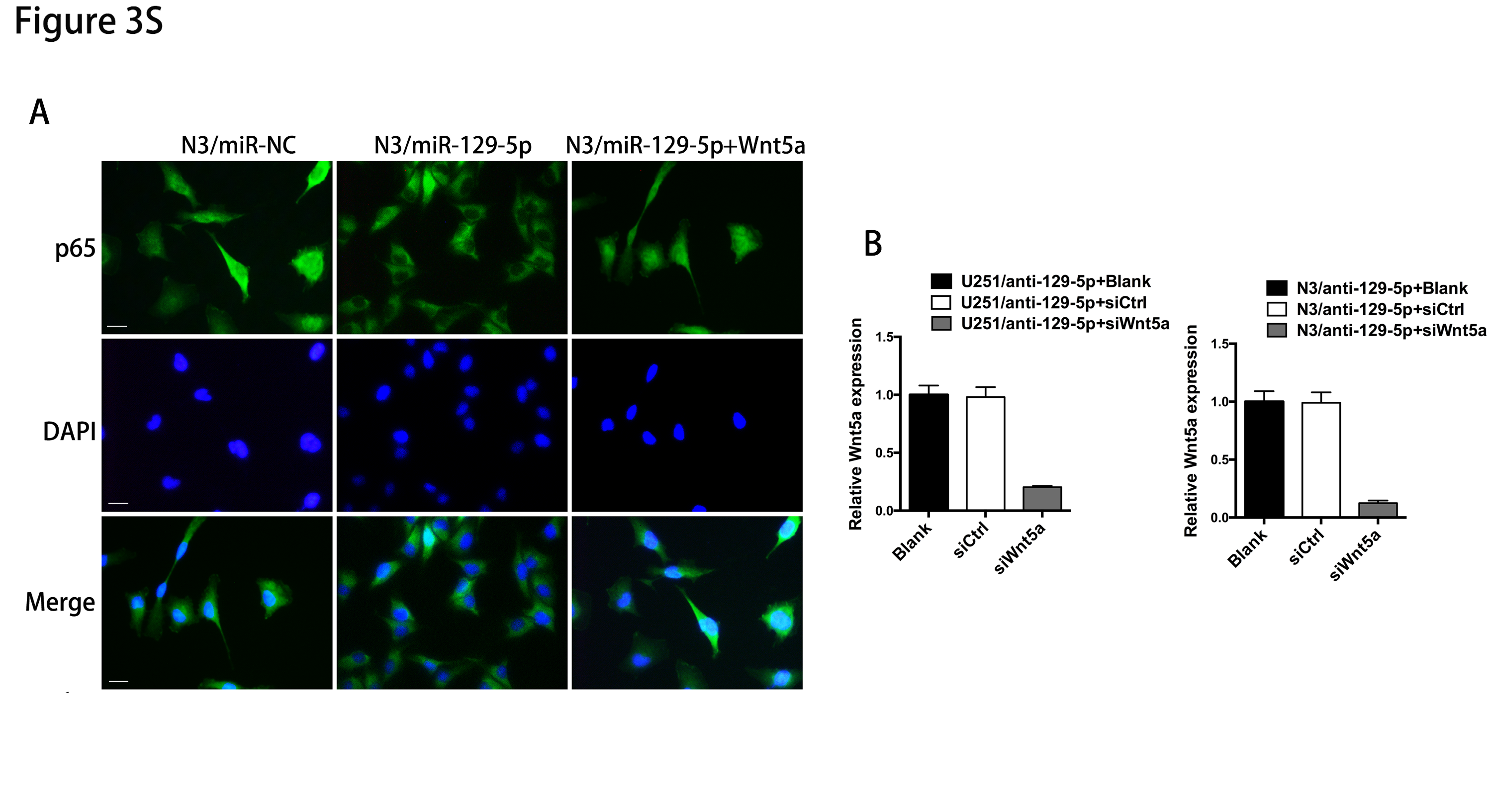


**Supplementary Figure 4 (Related to Figure 7).**

A, RT-qPCR analysis of miR-129-5p in N3 and U251 cells transfected with a lentiviral pCDH vector(control) or pCDH miR-129-5p expressing vector (pCDH miR-129-5p). U6 RNA served as the loading control.


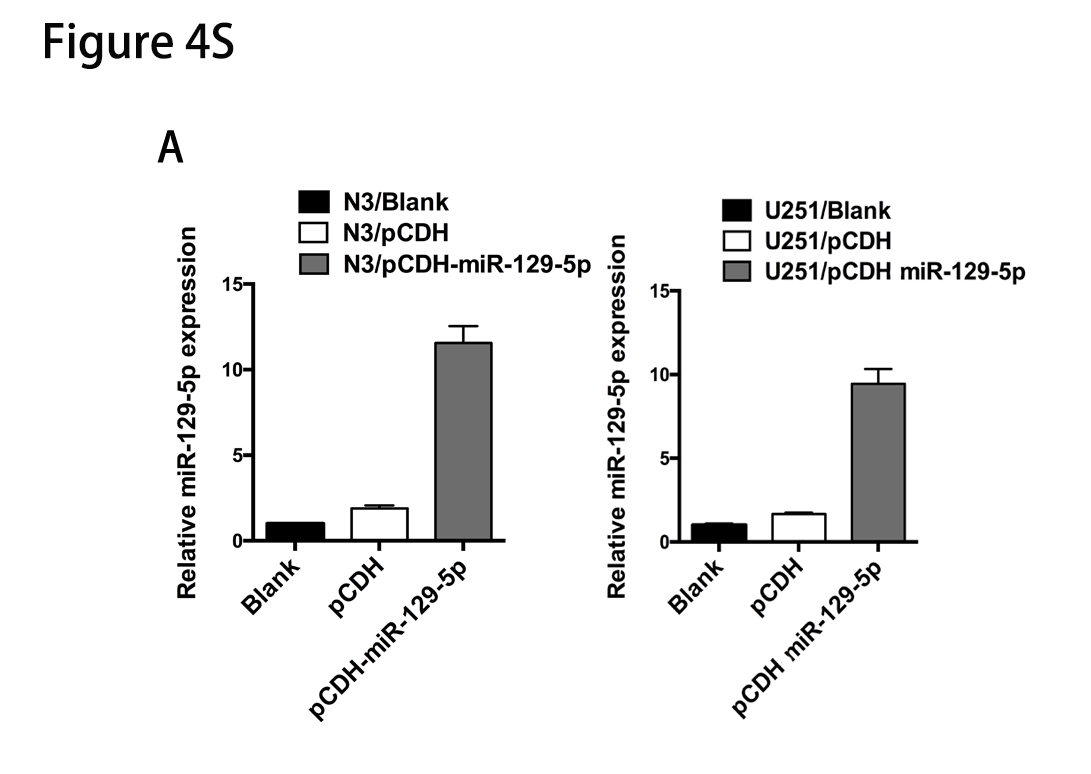


**Supplementary Table 1.** Univariate and multivariate analysis of malignant gliomas in the Rembrandt dataset

| Factor | Univariate | | Multivariate | |
| --- | --- | --- | --- | --- |
|  | ***P* value** | **HR** | ***P* value** | **HR** |
| Grade |  |  |  |  |
| Malignancy progress | <0.0001 | 1.86972 | <0.0001 | 1.51412 |
| Subtype |  |  |  |  |
| Classical | Ref. | Ref. | Ref. | Ref. |
| Mesenchymal | 0.18991 | 0.8160 | 0.0445 | 0.72808 |
| Neural | 0.00189 | 0.5372 | 0.1921 | 0.75189 |
| Proneural | <0.0001 | 0.2860 | <0.0001 | 0.36661 |
| Wnt5a |  |  |  |  |
| Increasing level | 0.035 | 1.13919 | 0.3642 | 1.06553 |

**Supplementary Table 2.** Univariate and multivariate analysis of malignant gliomas in the CGGA array dataset

| Factor | Univariate | | Multivariate | |
| --- | --- | --- | --- | --- |
|  | ***P* value** | **HR** | ***P* value** | **HR** |
| Age |  |  |  |  |
| Increasing years | <0.0001 | 1.041226 | 0.00259 | 1.021188 |
| Grade |  |  |  |  |
| Malignancy progress | <0.0001 | 2.9211 | <0.0001 | 2.545539 |
| Subtype |  |  |  |  |
| Classical | Ref. | Ref. | Ref. | Ref. |
| Mesenchymal | 0.918 | 1.02854 | 0.98544 | 0.994954 |
| Neural | <0.0001 | 0.29453 | 0.59581 | 0.833515 |
| Proneural | <0.0001 | 0.28016 | 0.03515 | 0.512969 |
| Wnt5a |  |  |  |  |
| Increasing level | 0.00014 | 1.36416 | 0.96356 | 0.995833 |
